# Combined Effects of Proton Radiation and Simulated Microgravity on the Cell Viability and ALP Activity of Murine Osteoblast Cells

**DOI:** 10.3389/fpubh.2021.759236

**Published:** 2021-11-30

**Authors:** Liqiu Ma, Fuquan Kong, Yihao Gong, Qiaojuan Wang, Jiancheng Liu, Li Sui

**Affiliations:** ^1^Department of Nuclear Physics, China Institute of Atomic Energy, Beijing, China; ^2^National Innovation Center of Radiation Application, Beijing, China

**Keywords:** proton radiation, microgravity, bone function, transcriptome, combined effects

## Abstract

Proton radiation (PR) and microgravity (μ*G*) are two key factors that impact living things in space. This study aimed to explore the combined effects of PR and simulated μ*G* (Sμ*G*) on bone function. Mouse embryo osteoblast precursor cells (MC3T3-E1) were irradiated with proton beams and immediately treated with Sμ*G* for 2 days using a three-dimensional clinostat. All samples were subjected to cell viability, alkaline phosphatase (ALP) activity and transcriptome assays. The results showed that cell viability decreased with increasing doses of PR. The peak ALP activity after PR or Sμ*G* alone was lower than that obtained with the non-treatment control. No difference in cell viability or ALP activity was found between 1 Gy PR combined with Sμ*G* (PR-Sμ*G*) and PR alone. However, 4 Gy PR-Sμ*G* resulted in decreased cell viability and ALP activity compared with those obtained with PR alone. Furthermore, Gene Ontology analysis revealed the same trend. These results revealed that PR-Sμ*G* may lead to reductions in the proliferation and differentiation capacities of cells in a dose-dependent manner. Our data provide new insights into bone-related hazards caused by multiple factors, such as PR and μ*G*, in the space environment.

## Introduction

With the comprehensive development of manned space exploration missions (such as manned missions to the Moon and manned missions to Mars), more astronauts need to be sent into space. Astronauts on space missions will experience space environmental stressors, such as continuous microgravity (μ*G*) and uninterrupted doses of ionizing radiation (1). The impact of the space environment on astronauts is directly related to their own health and whether the space mission can be successfully completed. Therefore, it is necessary to perform a risk assessment of the space environment for astronauts.

The risks of the μ*G* environment to astronauts' health cannot be ignored. Long-term space flight causes a variety of physiological and pathological changes in the body of astronauts, such as bone loss, decreased immune function, and muscle atrophy. One of the most obvious is the decrease in bone density caused by space μ*G*. A recent study conducted a systematic retrospective analysis of the bone density of 148 individuals who had performed space missions and found that the changes in bone density values caused by space microgravity depend on the skeletal-site position relative to the gravitational vector. For example, compared with the upper limbs and thoracic vertebrae, the bone density of pelvis, lumbar vertebrae and lower limbs has decreased more severely (2). The average bone loss of 1% per month during space flight is a serious threat to the health of astronauts (3), and the main cause of space bone loss is decreased bone formation and not bone resorption (4, 5). In addition to the effects of μ*G* on astronauts, space radiation also poses a threat to their health. Space agencies have stated that the maximum allowable dose for an astronaut over a lifetime is approximately 1 Sv (6). Radiobiology studies have shown that ionizing radiation damage is divided into physical damage directly caused by charged particles to biologically active molecules and damage indirectly caused by free radicals generated by the reaction of charged ions with water (7). At the molecular level, ionizing radiation can induce gene mutations, gene expression changes, DNA methylation, and protein expression (8–11). At the cellular level, ionizing radiation damage mainly includes decreased cell survival, cell cycle arrest, and chromosomal aberrations (12). At the human level, this damage is not only associated with a higher cancer risk (13) but can also cause temporary or permanent damage to organs such as the cardiovascular system (14), central nervous system (15), and eyes (16) and can even endanger the life of astronauts in severe cases. However, the influence of space radiation on bone remains unclear.

Furthermore, the combined effect of space radiation and μ*G* has been widely considered. Horneck et al. showed that various organisms are irradiated before space flight to test the influence of μ*G* on the repair of radiation-induced DNA damage, but again, no significant differences were detected between space and ground (17). It has been reported that the presence of μ*G* enhances the DNA damage, mutagenic effects and chromosome aberrations induced by radiation (18–20). However, the combined effects of radiation and μ*G* on bone cells are not well-understood.

Protons are one of the main components of space radiation and exhibit the largest proportion and highest flux (21). In spacecraft and space stations, high-energy protons can directly pass through the shielding layer. The nuclear reaction of high-energy heavy ions in galaxy cosmic rays with the shielding material will also produce a large number of secondary protons (22, 23). Obtaining more basic data based on the combination of proton radiation (PR) and simulated μ*G* (Sμ*G*) is necessary to reasonably evaluate the effects of the space environment. In this study, we evaluated the viability and alkaline phosphatase (ALP) activity of osteoblast MC3T3-E1 cells to investigate the biological effect of PR combined with Sμ*G* (PR-Sμ*G*).

## Materials and Methods

### Cell Culture

Mouse embryo osteoblast precursor MC3T3-E1 cells were purchased from Institute of Basic Medical Sciences Chinese Academy of Medical Sciences. MC3T3-E1 cells were cultured in α-MEM (HyClone, Logan, UT, USA). All growth media contained 10% fetal bovine serum (HyClone), penicillin (50 units/ml), and streptomycin (50 μg/ml). For the examination of ALP activity, untreated control cells and PR- and/or Sμ*G*-treated cells were plated in 12-well plates and incubated in osteogenic medium consisting of growth media supplemented with 50 μg/ml L-ascorbic acid (vitamin C), 10 mM β-glycerophosphate and 100 nM dexamethasone. All cells were maintained at 37°C in an incubator containing 5% CO_2_.

### Experimental Design

**Figure 1:**
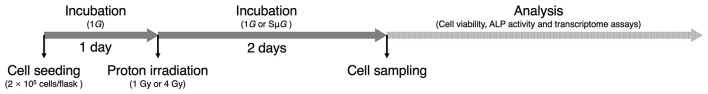
Experimental schedule. After 1 day of incubation, the cells were exposed to proton beams, and 1*G* or Sμ*G* was immediately applied for 2 days. All the samples were subjected to subsequent analysis.

Mouse embryo osteoblast precursor cells (MC3T3-E1) were plated in a T25 flask at a density of 2 × 10^5^ cells/flask. After 24 h of incubation, the cells were exposed to 1 Gy (astronaut career limit dose) (6) or 4 Gy (half lethal dose of bone marrow cells) (24) proton beams (22 MeV, 0.8 Gy/min) at the Beijing Tandem Accelerator Nuclear Physics National Laboratory, and Sμ*G* (~10^−3^ *G*) was immediately applied for 2 days using a three-dimensional clinostat (Gravite, Space Bio-Laboratories Co., Ltd., Hiroshima, Japan). All the samples were subjected to cell viability, ALP activity and transcriptome assays (Figure 1).

### Cell Viability Assay

A Cell Counting Kit-8 (CCK-8) assay (DOJINDO, Kumamoto, Japan) was used to estimate the cell viability of osteoblasts after PR and/or Sμ*G* treatments. Briefly, untreated control cells and PR- and/or Sμ*G*-treated cells were plated in 96-well plates at a density of 4 × 10^3^ cells/well, and 6 replicate wells of each cell group were included in the plates. The cells were cultured for 0, 12, 36, 60, 84 h. At each end point, 10 μl of CCK-8 reagent was added to the corresponding wells, and the plates were incubated for 2 h at 37°C in an incubator containing 5% CO_2_. The absorbance values at 450 nm were read using an automated microplate reader (Beijing Perlong New Technology Co., Ltd., Beijing, China). The cell viability (fold change) of each group at different time points was normalized by the cell viability on Day 0 (0 h).

### Cell ALP Activity Assay

Untreated control cells and PR- and/or Sμ*G*-treated cells were plated in 12-well plates at a density of 2 × 10^4^ cells/well and cultured for 4, 7, and 10 days. The medium was changed every 3 days. At each end point, the cells were washed three times with PBS and suspended in 200 μl of Triton X-100 lysis buffer (Beijing Leagene Biotechnology Co., Ltd., Beijing, China) for 10 s. The homogenate was centrifuged at 12,000 × *g* for 10 min, and the supernatant was used for subsequent BCA protein and ALP activity assays. Total protein was quantified using a Pierce BCA Protein Assay Kit according to the manufacturer's instructions (Pierce Biotechnology, Rockford, IL, USA). ALP activities in the cultured cells were determined by measuring released *p*-nitrophenyl (FUJIFILM Wako Pure Chemical Co., Osaka, Japan). After 15 min of incubation at 37°C, the absorbance of *p*-nitrophenyl at 405 nm was measured using an automated microplate reader (Beijing Perlong New Technology Co., Ltd., Beijing, China). The relative ALP activity was defined as the concentration of released *p*-nitrophenyl per minute per μg of protein.

### RNA Isolation and Library Preparation

Total RNA was extracted using TRIzol reagent according to the manufacturer's protocol. The RNA purity and quantification were evaluated using a NanoDrop 2000 spectrophotometer (Thermo Scientific, USA). RNA integrity was assessed using the Agilent 2100 Bioanalyzer (Agilent Technologies, Santa Clara, CA, USA). Libraries were then constructed using the TruSeq Stranded mRNA LT Sample Prep Kit (Illumina, San Diego, CA, USA) according to the manufacturer's instructions.

### RNA Sequencing and Transcriptome Analysis

The libraries were sequenced on an Illumina HiSeq X Ten platform, and 150-bp paired-end reads were generated. Approximately 50 M raw reads were generated from each sample. The raw data (raw reads) in FASTQ format were first processed using Trimmomatic (25), and the low-quality reads were removed to obtain clean reads. The deep sequencing data from the RNA sequencing analyses have been deposited under accession number PRJNA754381 in the NCBI (National Center for Biotechnology Information) Sequence Read Archive.

The clean reads were mapped to the mouse genome (GRCm38) using HISAT2 (26). The FPKM (27) value of each gene was calculated using Cufflinks (28), and the read counts of each gene were obtained with HTSeq-count (29). Differential expression analysis was performed using the DESeq (2012) R package. To investigate the profile of genes that exhibit changes in expression, a screening of genes was performed based on a threshold of *p*-value < 0.05 and a fold change > 2 or fold change < 0.5. A hierarchical cluster analysis of differentially expressed genes (DEGs) was performed to demonstrate the expression pattern of genes in different groups. To gain a general idea of the biological functions of the DEGs, a gene ontology (GO) enrichment analysis was performed using R based on the hypergeometric distribution. A false discovery rate (FDR) <0.05 served as the threshold to select the GO categories that were significantly enriched.

### Statistical Analysis

The statistical significance of the differences was tested using Student's *t*-test. The differences between the means were considered statistically significant if *p* < 0.05.

## Results

### Characterization of MC3T3-E1 Cell Viability After PR-Sμ*G* Treatment

**Figure 2:**
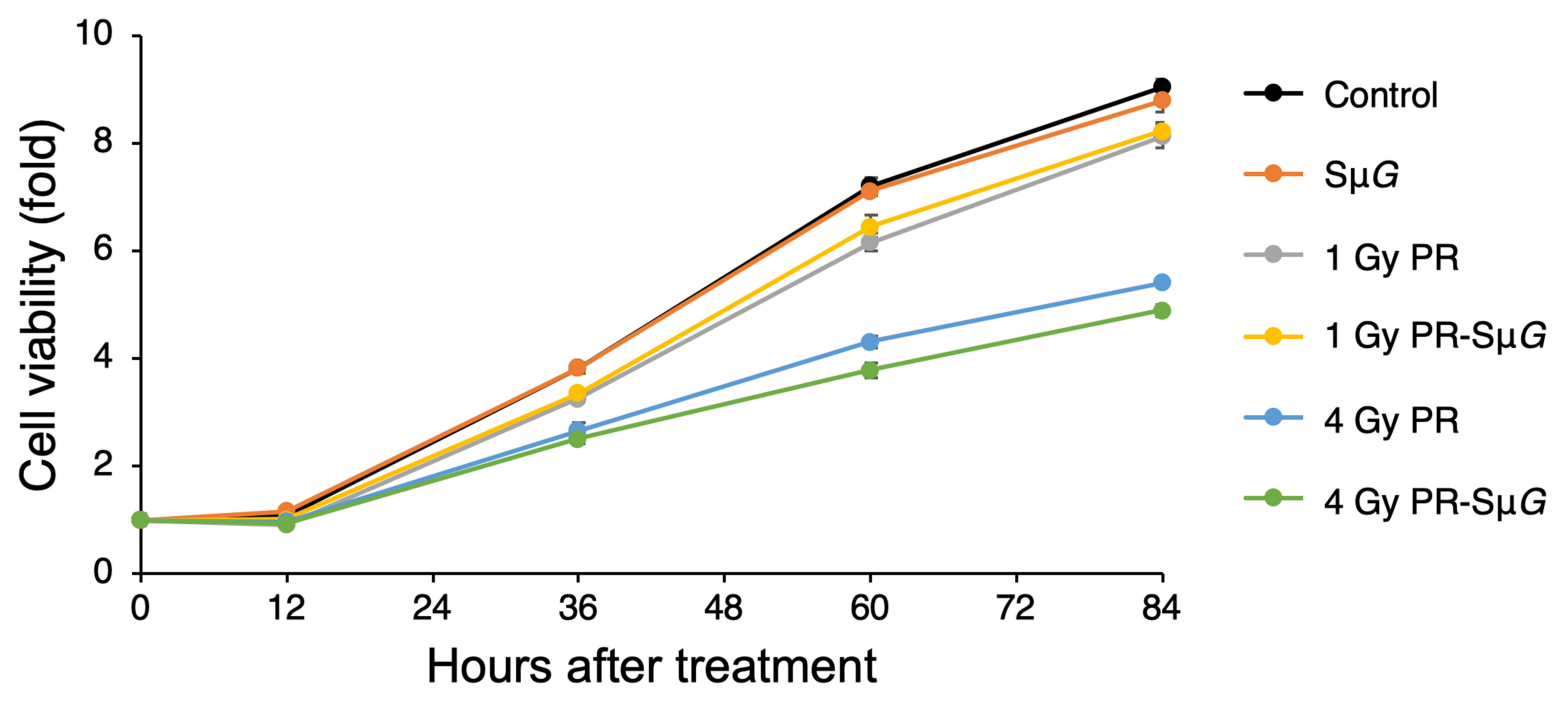
Time course of the effects of Sμ*G*, PR, and their combination on cell viability. Symbols: control (black closed circles), Sμ*G* (orange closed circles), 1 Gy PR (gray closed circles), 1 Gy PR-Sμ*G* (yellow closed circles), 4 Gy PR (blue closed circles), and 4 Gy PR-Sμ*G* (green closed circles). The bars indicate the standard errors calculated using data from three independent trials.

We examined the cell viability of MC3T3-E1 cells after PR-Sμ*G* treatment. MC3T3-E1 cells were irradiated with 1 Gy or 4 Gy proton beams, and Sμ*G* was then immediately applied for 2 days using a three-dimensional clinostat. After these treatments, a cell viability analysis was performed using the CCK-8. No detectable difference in cell viability was found between the control and Sμ*G* groups (Figure 2). Cell viability decreased with increasing doses of PR alone. No difference in cell viability was detected between 1 Gy PR-Sμ*G* and 1 Gy PR alone after 36 hours, whereas the 4 Gy PR-Sμ*G* treatment resulted in significantly lower viability than that obtained with the 4 Gy PR alone (*p* < 0.05).

### Evaluation of the ALP Activity of MC3T3-E1 Cells After PR-Sμ*G* Treatment

**Figure 3:**
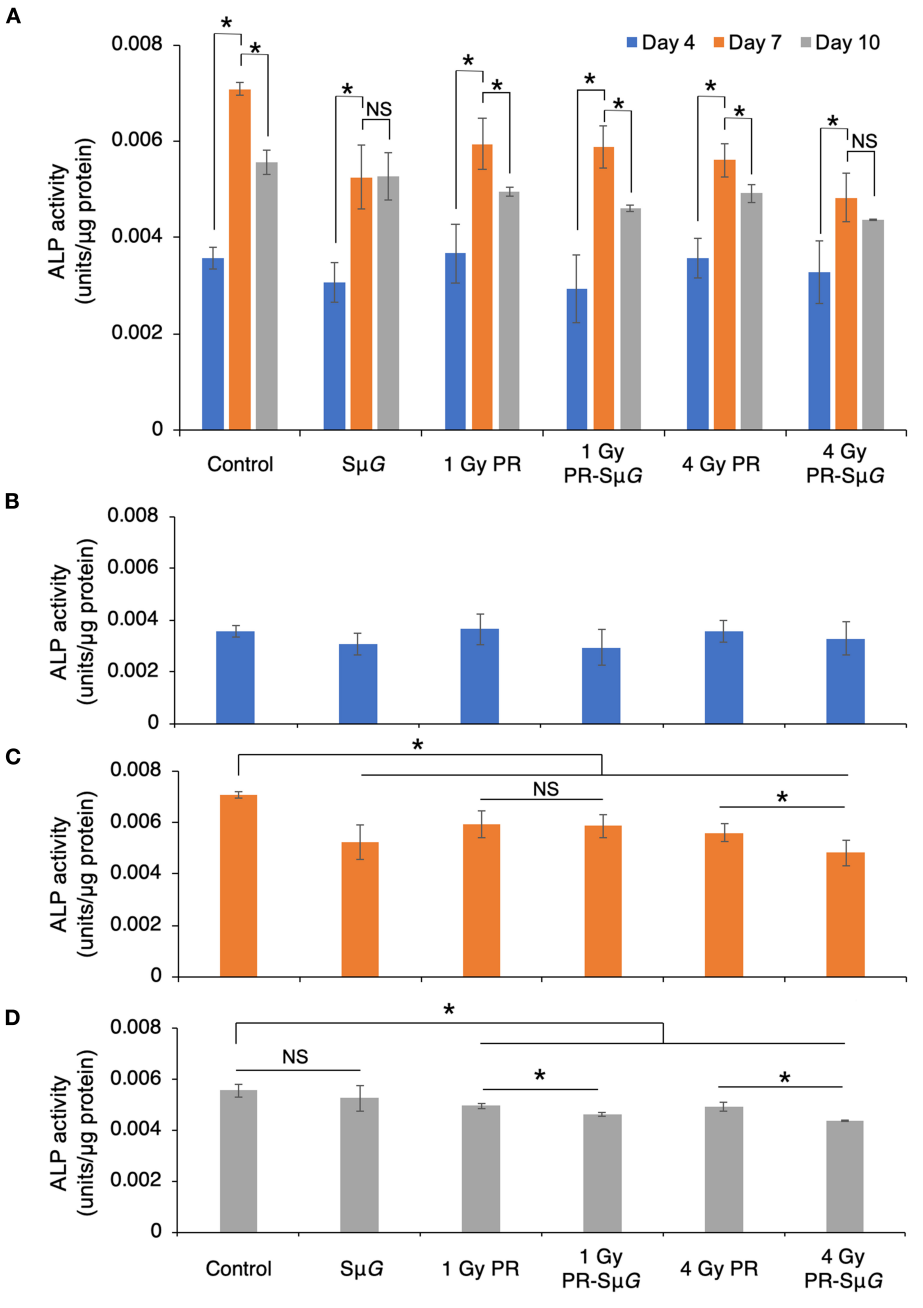
Effects of Sμ*G*, PR, and their combination on ALP activity. **(A)** Time course of ALP activity. ALP activity at day 4 **(B)**, day 7 **(C)**, and day 10 **(D)** posttreatment. The mean values with standard errors are shown. *p < 0.05, NS indicates not significant (*p* > 0.05) (Student's *t*-test). Symbols: day 4 (blue column), day 7 (orange column), and day 10 (gray column). The bars indicate the standard errors calculated using data from three independent trials.

To further understand the effect of PR-Sμ*G* treatment on the function of osteoblasts, we evaluated the ALP activity of MC3T3-E1 cells on days 4, 7, and 10 after PR and/or Sμ*G* treatment. ALP activity was significantly increased in all the groups from days 4 to 7 after treatment. The highest ALP activity was observed at day 7 in all the groups. ALP activity in the Sμ*G* and 4 Gy PR-Sμ*G* groups did not significantly change from day 7 to day 10, but the other four groups showed a significant decrease during this period (Figure 3A). At day 4 after treatment, no significant difference in ALP activity was found among the groups (Figure 3B). At day 7 after treatment, ALP activity was inhibited in all five treatment groups compared with the control group. Although no significant difference in ALP activity was found between the 1 Gy PR and 1 Gy PR-Sμ*G* groups, that of the 4 Gy PR-Sμ*G* group was significantly lower than that of the 4 Gy PR group (Figure 3C). At day 10 after treatment, although no significant difference in ALP activity was found between Sμ*G* alone and the control groups, that of the PR-Sμ*G* group was significantly lower than that of the PR group (Figure 3D). Therefore, the Sμ*G* alone and PR alone can each inhibit ALP activity, and the PR-Sμ*G* treatment can further reduce the activity of ALP.

### Transcriptome Features of MC3T3-E1 Cells After PR-Sμ*G* Treatment

In this study, 20 samples from the five experimental groups (three independent biological replicates of each group) and the control group (five replicates) were sequenced, and a total of 936,781,862 high-quality reads were obtained from the experimental groups (Supplementary Table 1). A nucleotide composition analysis showed that the total GC content of the transcriptome data was 52.40%. The analysis of the base composition and quality showed that the base composition of the original sequencing data was in good condition. For each replicate, 98.28-98.85% of the reads could be mapped to the mouse genome (GRCm38) using HISAT2, and among these mapped reads, 92.11-93.01% achieved a unique match. All sequences covered the reference genes evenly, which showed that the overall quality of the sequences was good and that the sequencing data were random and could be used for subsequent analysis.

To clarify the mechanism underlying the characteristic changes after PR-Sμ*G* treatment, the transcriptome features of PR and/or Sμ*G* treatment were assessed by RNA-seq analysis, and an osteogenesis-related GO enrichment analysis was performed (Table 1). The GO analysis of the DEGs identified from the comparison of 1 Gy PR vs. 1 Gy PR*-*Sμ*G* resulted in the selection of only the bone morphogenesis-related category (GO:0060349). In contrast, three osteoblast proliferation- or differentiation-related GO categories (GO:0033690, GO:0045667, and GO:0045779) were found to be changed in the comparison of 4 Gy PR vs. 4 Gy PR*-*Sμ*G*. We did not find any GO category from the comparison of Sμ*G* vs. 1 Gy PR*-*Sμ*G*, whereas four osteogenesis-related GO categories were found to differ between the Sμ*G* and 4 Gy PR*-*Sμ*G* groups.

**Table 1:** List of gene function annotations related to PR and/or Sμ*G*.

	**GO ID**	**GO term**	**FDR value**
**vs. 1 Gy PR-Sμ** ***G***			
1 Gy PR	GO:0060349	Bone morphogenesis	0.020898789
Sμ*G*	N/A	N/A	N/A
**vs. 4 Gy PR-Sμ** ***G***			
4 Gy PR	GO:0033690	Positive regulation of osteoblast proliferation	0.029716189
	GO:0045667	Regulation of osteoblast differentiation	0.036043093
	GO:0045779	Negative regulation of bone resorption	0.036043093
Sμ*G*	GO:0030500	Regulation of bone mineralization	0.004557925
	GO:0030282	Bone mineralization	0.007654929
	GO:0060349	Bone morphogenesis	0.008773026
	GO:0045669	Positive regulation of osteoblast differentiation	0.022438056

*N/A, not applicable*.

## Discussion

The effect of the space environment on cell viability is due to many factors, such as space radiation and μ*G*. Previous studies have shown that short-duration space travel can cause significant changes in the number and size of osteoblasts (30). Consistently, our experimental data suggest that PR-Sμ*G* also inhibits cell viability (Figure 2). Furthermore, treatment with PR alone but not Sμ*G* alone could induce the inhibition of cell activity. Therefore, compared with μ*G* factors, radiation factors may be an important factor affecting osteoblast viability. In contrast, a large solar proton event (SPE) could be encountered during a long-duration deep-space flight or during long-term missions such as landing on Mars. The risk to astronauts of high-dose proton irradiation in the SPE cannot be ignored. Our experimental results show that a high dose (4 Gy) of PR combined with Sμ*G* has a stronger inhibitory effect on cell viability than PR alone. Although a previous study showed that the radiation-induced DNA damage repair of fibroblasts is not affected by μ*G* (17), our results suggest that the radiation-induced osteoblast DNA damage repair ability may be affected by μ*G*. These results indicate that PR combined with Sμ*G* may induce an additional inhibitory effect on osteoblastic viability, and the level of inhibition increases with increasing radiation dose.

ALP is a glycoprotein that can catalyse the hydrolysis of monophosphoric acid in alkaline environments. This protein can hydrolyse inorganic phosphate, increase the concentration of local inorganic phosphate, promote bone mineralization, and provide conditions for the formation of hydroxyapatite crystallization in bone tissue, which results in initiation of the processes of extracellular matrix mineralization and calcium and phosphorus deposition (31, 32). Therefore, ALP is an important marker of the differentiation and maturation of osteoblasts, and its activity is one of the important indicators of osteocyte function, which can indirectly reflect the function of osteoblasts (33). The effect of the space environment on the bone formation process has been confirmed by space missions (Euromir 95) (34). This study revealed the trends in the change in bone metabolic markers during the 180-day stay on the Mir space station. The results showed that the concentration of the bone formation marker ALP decreased sharply from 0 to 50 days during space flight, whereas the concentration of the bone absorption marker D-pyridinoline increased. The above experimental results prove that the differentiation function of bone cells is reduced in the space environment. Because osteoblasts secrete ALP during differentiation, once osteoblasts differentiate into osteocytes, these cells will not secrete ALP (35). Our results showed that the ALP levels were low 4 days after treatment, which indicated that the cells were in prophase of osteoblastic differentiation (Figure 3A). The ALP level reached the highest level after 7 days, which indicated the highest differentiation level of osteoblasts. After 10 days, ALP gradually decreased, indicating that some osteoblasts had differentiated into osteocytes. Our experimental data indicated that PR and/or Sμ*G* inhibited ALP activity at the highest differentiation phase of osteoblasts (day 7, Figure 3C). Consistent with the cell viability results, only a high dose (4 Gy) of PR combined with Sμ*G* significantly inhibited ALP activity compared with the results obtained with PR alone. These results suggest that a higher dose of PR combined with Sμ*G* may produce an additional ALP activity inhibitory effect on osteoblasts due to the lower cell viability. At day 10 after treatment, no significant difference in ALP activity was found between the Sμ*G* group and the control group, whereas the ALP activity level in the irradiation groups was significantly lower (Figure 3D). The results indicated that the ALP activity of the Sμ*G* alone group recovered completely, and the recovery ability of the other groups was weak. Compared with that in the PR group, the ALP activity in the PR-Sμ*G* group was lower, indicating that the recovery of osteoblast differentiation function was more difficult under the environment induced by the combined effects of PR-Sμ*G* treatment.

The cell viability and function of osteoblasts showed differences between the 4 Gy PR and 4 Gy PR-Sμ*G* groups but not between the 1 Gy PR and 1 Gy PR-Sμ*G* groups. Our transcriptome analysis data strongly support this result (Table 1). Only the comparison of 4 Gy PR vs. 4 Gy PR-Sμ*G* revealed a change in GO:0033690, which is related to activating or increasing the rate or extent of osteoblast proliferation, whereas the comparison of 1 Gy PR vs. 1 Gy PR-Sμ*G* showed no significant change in GO:0033690. This result is consistent with the cell viability results. In addition, only the comparison of 4 Gy PR vs. 4 Gy PR-Sμ*G* showed changes in GO:0045667, which is related to modulating the frequency, rate or extent of osteoblast differentiation, whereas 1 Gy PR vs. 1 Gy PR-Sμ*G* showed no significant changes in GO:0045667. This result was consistent with the results from the analysis of cell ALP activity. Together, these results suggest that PR-Sμ*G* treatment has the ability to alter genes involved in osteoblast proliferation and differentiation and that the expression of these genes ultimately leads to changes in cell viability and ALP activity.

One limitation of this experiment lies in the use of only ALP activity assay to explore the possible effect of PR and/or Sμ*G* on bone function; however, the ALP expression still shows similar trends with GO term among groups. These results provide valuable reference evidence for further studies using other bone functional markers and even the rodent hindlimb unloading model on the combined biological effects of PR-Sμ*G*.

In conclusion, our results suggest that PR-Sμ*G* may exert additional inhibitory effects on the proliferation and differentiation of osteoblasts in a dose-dependent manner. Radiation may be an important factor affecting the recovery of the differentiation ability of osteoblasts, regardless of treatment with PR alone or PR-Sμ*G*. Our data provide new insights for understanding bone-related hazards caused by multiple factors, such as PR and μ*G*, in the space environment.

## Data Availability Statement

The datasets presented in this study can be found in online repositories. The names of the repository/repositories and accession number(s) can be found at: https://www.ncbi.nlm.nih.gov/, PRJNA754381.

## Author Contributions

LM and LS designed research. LM, FK, YG, JL, and QW performed the research. LM analyzed data and drafted the manuscript. LM and LS revised the manuscript. All authors have read and agreed to the final manuscript.

## Funding

This research was funded by the Continuous Basic Scientific Research Project (grant number WDJC-2019-11).

## Conflict of Interest

The authors declare that the research was conducted in the absence of any commercial or financial relationships that could be construed as a potential conflict of interest.

## Publisher's Note

All claims expressed in this article are solely those of the authors and do not necessarily represent those of their affiliated organizations, or those of the publisher, the editors and the reviewers. Any product that may be evaluated in this article, or claim that may be made by its manufacturer, is not guaranteed or endorsed by the publisher.
