# Supplementary material for: Combined Effects of Proton Radiation and Simulated Microgravity on the Cell Viability and ALP Activity of Murine Osteoblast Cells
Source: Front Public Health. 2021 Nov 30;9:759236. doi: 10.3389/fpubh.2021.759236 (PMC8669367; doi:10.3389/fpubh.2021.759236)
Supplement: Supplementary file 1 [file Table_1.DOCX]

Supplementary Material

# Supplementary Tables

Table S1. Statistical results of reads and reference genome comparison rates.

| **Table S1. Statistical results of reads and reference genome comparison rates** | | | | | |
| --- | --- | --- | --- | --- | --- |
|  | Duplicate samples | Total reads | Total mapped reads (percentage) | Multiple mapped reads (percentage) | Uniquely mapped reads (percentage) |
| Control | No. 1 | 47583296 | 46762825(98.28%) | 2928535(6.15%) | 43834290(92.12%) |
|  | No. 2 | 47565200 | 46955336(98.72%) | 2904736(6.11%) | 44050600(92.61%) |
|  | No. 3 | 41375282 | 40858592(98.75%) | 2501531(6.05%) | 38357061(92.71%) |
|  | No. 4 | 43595926 | 43034395(98.71%) | 2645648(6.07%) | 40388747(92.64%) |
|  | No. 5 | 47859092 | 47252657(98.73%) | 2923093(6.11%) | 44329564(92.63%) |
| 1 Gy PR | No. 1 | 47494858 | 46908074(98.76%) | 2864015(6.03%) | 44044059(92.73%) |
|  | No. 2 | 48544642 | 47922507(98.72%) | 2924544(6.02%) | 44997963(92.69%) |
|  | No. 3 | 50120214 | 49434001(98.63%) | 2984329(5.95%) | 46449672(92.68%) |
| 4 Gy PR | No. 1 | 47406534 | 46827634(98.78%) | 3069835(6.48%) | 43757799(92.30%) |
|  | No. 2 | 48017080 | 47444309(98.81%) | 3110385(6.48%) | 44333924(92.33%) |
|  | No. 3 | 45652882 | 45058899(98.70%) | 2972509(6.51%) | 42086390(92.19%) |
| 1 Gy  PR-Sμ*G* | No. 1 | 47114028 | 46500278(98.70%) | 2756920(5.85%) | 43743358(92.85%) |
|  | No. 2 | 49355106 | 48786925(98.85%) | 2883139(5.84%) | 45903786(93.01%) |
|  | No. 3 | 46248922 | 45658826(98.72%) | 2686080(5.81%) | 42972746(92.92%) |
| 4 Gy  PR-Sμ*G* | No. 1 | 47796852 | 47145594(98.64%) | 2963842(6.20%) | 44181752(92.44%) |
|  | No. 2 | 45558586 | 44956675(98.68%) | 2794698(6.13%) | 42161977(92.54%) |
|  | No. 3 | 43921150 | 43347717(98.69%) | 2662109(6.06%) | 40685608(92.63%) |
| Sμ*G* | No. 1 | 45768446 | 45196268(98.75%) | 2885420(6.30%) | 42310848(92.45%) |
|  | No. 2 | 49147204 | 48531144(98.75%) | 3134728(6.38%) | 45396416(92.37%) |
|  | No. 3 | 46656562 | 46066981(98.74%) | 3093809(6.63%) | 42973172(92.11%) |
